# Essential Updates 2018/2019: Essential Updates for esophageal cancer surgery

**DOI:** 10.1002/ags3.12319

**Published:** 2020-02-18

**Authors:** Yasuyuki Seto

**Affiliations:** ^1^ Department of Gastrointestinal Surgery Graduate School of Medicine The University of Tokyo Tokyo Japan

**Keywords:** centralization, esophageal cancer, hospital volume, minimally invasive esophagectomy, robot

## Abstract

Key papers to treatment of esophageal cancer surgery and reduction of postoperative complications after esophagectomy published between 2018 and 2019 were reviewed. Within this review there was a focus on minimally invasive esophagectomy (MIE), robot‐assisted MIE (RAMIE), and centralization to high‐volume center. Advantages of MIE, irrespectively of hybrid or total MIE, to prevent postoperative complications, especially pneumonia, were shown in comparison to open procedure. However, whether total MIE has evident effects or not, as compared to hybrid MIEs, still remains unclear. Differences between RAMIE and MIE were reported to be marginal, though the advantage of lymphadenectomy, especially along recurrent laryngeal nerve, has been suggested. Centralization to high‐volume center evidently benefits esophageal cancer patients by improving short‐term outcomes. The definition of high‐volume center has not been established yet, though institutional structure and quality are thought to be important. Transmediastinal esophagectomy, currently developed, has a potential to be one radical option of MIE for esophageal cancer.

## Abstract 2

The key papers to treatment of esophageal cancer surgery and reduction of the postoperative complications published worldwide in the period between 2018 and 2019 were reviewed.

## 1 INTRODUCTION

Surgery remains a mainstay of treatment for esophageal cancer worldwide. It is, however, one of the most invasive procedures and is associated with high morbidity. Postoperative complications, especially pulmonary complications such as pneumonia, were reported to reduce the survival rate.^1^, ^2^, ^3^ Therefore, the prevention of postoperative complications is the urgent and most important issue. The key papers to treatment of esophageal cancer surgery and reduction of the postoperative complications published worldwide in the period between 2018 and 2019 were reviewed. With a focus on minimally invasive esophagectomy (MIE), robot‐assisted procedure, and the centralization to high‐volume center, this review evaluates their effects and significance to reducing morbidity, especially pulmonary complications.

## 2 MINIMALLY INVASIVE ESOPHAGECOMY (MIE)

The first MIE was reported in 1992 by Cuschieri et al.^4^ In those five patients, video‐assisted thoracoscopic surgery for esophagectomy and laparotomy were used. The combinations of thoracoscopy and laparotomy, such as in the report by Cushcieri, are regarded as hybrid MIE. Total MIE, i.e., the procedures performed by the combination of thoracoscopy and laparoscopy, was first reported by Luketish et al.^5^ Gottlieb‐Vedi et al have defined the MIE as follows. Total MIE was defined as surgery in which there was no thoracotomy or laparotomy performed. Hybrid MIE is defined as either thoracotomy with laparoscopy; laparotomy with thoracoscopy; or laparotomy with mediastinoscopy.^6^

As for the hybrid MIE, two reports of thoracoscopy combined with laparoscopy or laparotomy (each number was not shown) based on big data were published in Japan. Based on the latest analysis of 24 233 esophagectomies performed between 2012 and 2016 and registered in the Japanese National Clinical Database (NCD),^7^ Yoshida et al showed its significant superiority to open esophagectomy in terms of postoperative morbidities and surgery‐related mortality.^8^ Sakamoto et al analyzed the propensity matching that generated 4572 pairs among 14 880 patients in the Japanese inpatients database. The significantly lower incidences of in‐hospital mortality, surgical site infection, and anastomotic leakage in the hybrid MIE group were also shown in comparison to the open esophagectomy group.^9^ These two studies were retrospective, but it is important to consider that they were based on a large cohort, and no useful randomized controlled trial has reported on this topic to date. Two propensity score‐matched analyses from a single institution study also showed less respiratory complications^10^ and lower CRP levels 11 in hybrid MIE as compared to open esophagectomy, respectively. Another retrospective single institution study demonstrated clear contribution of hybrid MIE to long‐term respiratory function after esophagectomy in comparison to open esophagectomy.^12^

A randomized controlled trial of hybrid MIE of laparoscopy with open right thoracotomy as compared to laparotomy was done under a multicenter and open‐label setting in France from 2009 through 2012 and has been reported.^13^ One hundred and three and 104 patients were assigned to hybrid and open groups, respectively. Significantly lower incidence of intraoperative and postoperative major complications, especially pulmonary ones, were found in the hybrid group.

Few comparative studies between total MIE and open procedure have been reported to date. A propensity score‐matched analysis of the NSQIP database from 2016 through 2017 compared the outcomes of total MIE to open procedures.^14^ The results of 161 pairs showed that overall complication rates were significantly less in MIE procedures, though the open procedure was associated with higher reported rates of abdominal and mediastinal lymphadenectomies. Table 1 summarizes the short‐term outcomes of MIE versus open procedures.

**Table 1:** short‐term outcomes of MIE vs Open

Author	MIE	Study design	No. patients	Superior information
Yoshida^8^	Hybrid; thoracoscopy	Japan national database (NCD) Retrospective	24 233	Most postoperative complications Pulmonary morbidity Surgery‐related mortality
Sakamoto^9^	Hybrid; thoracoscopy	Japan DPC database Propensity score matched	4572 pairs	Tracheotomy, unplanned intubation In‐hospital mortality
Chan^10^	Hybrid; thoracoscopy	Single institution Propensity score matched	345	Respiratory complications
Mariette^13^	Hybrid; laparoscopy	Multicenter RCT	207	Pulmonary complications
Naffouje^14^	Total	ACS NSQIP database Propensity score matched	161 pairs	Overall complications Pneumonia; not significant

Abbreviation: ACS NSQIP, American College of Surgeons National Surgical Quality Improvement Program.

As in Gottlieb‐Vedi's definition,^6^ there are several types of hybrid MIE. However, very few studies comparing outcomes among those hybrid MIE procedures and total MIE to hybrid MIEs have been shown to date. A prospective study with 25 cases each reported that total MIE seemed to be associated with a low incidence of complications such as pneumonia and wound infections as compared to hybrid MIE^15^; however, that study is too small in size to be included. Therefore, the results of the ROMIO study planned in the UK—a randomized controlled trial comparing open esophagectomy, hybrid MIE, and total MIE— are anticipated.^16^

In a short summary, the above‐mentioned papers suggested the advantages of MIE, irrespectively of hybrid or total MIE, to prevent postoperative complications, especially pneumonia; however, some disadvantages, such as longer operation time, 9, 10, 11, 14 were also reported. Whether total MIE has evident effects as compared to hybrid MIEs or not still remains unclear. In addition, regarding oncological aspects, some papers have already shown longer survival of hybrid MIE as compared to open procedures.^10^, ^11^, ^13^ Furthermore, long‐term results of above‐mentioned RCT^13^ showed better results for hybrid MIE that consisted of laparoscopy and open thoracotomy on health‐related quality of life (QOL) as compared to open procedures.^17^

As Gottlieb‐Vedi's definition included mediastinoscopic procedure,^6^ recently, the usefulness of transmediastinal approach using mediastinoscopy, i.e., less pulmonary complications and better QOL, was recently introduced.^18^ Transhiatal esophagectomy has been performed as a less invasive procedure, though it is regarded as less radical because of insufficient lymphadenectomy. Transmediastinal esophagectomy consists of the combination of the transhiatal and transcervical approaches, and was shown to enable the similar mediastinal lymphadenectomy to transthoracic approach.^19^ In that radical procedure, neither transthoracic approach nor one‐lung ventilation anesthesia are necessary. This approach has the potential to be one option as a radical surgical procedure.

## 3 ROBOT‐ASSISTED PROCEDURE

Robot‐assisted esophagectomy was firstly reported by Horgan et al.^20^ In that case, transhiatal approach was applied, in which the procedure of lymphadenectomy was thought to be insufficient as radical esophageal cancer surgery. Kernstine et al showed the first robotic, two‐stage, three‐field lymphadenectomy in 2004.^21^ In Japan, Hashizume played a pioneer role in this field^22^ and performed the first robot‐assisted esophagectomy for esophageal cancer on 15 May 2001 (personal communication). Robot‐assisted procedures for esophageal cancer have been performed as one of MIEs, therefore, usually abbreviated as RAMIE.

Many papers focusing on robot‐assisted procedures of treatment for esophageal cancer have been published from 2018 through 2019, because robots have been increasingly used in MIE. Among them, studies of comparison between RAMIE and open procedures were, however, few; just two papers. Hillegersberg et al showed less overall surgery‐related postoperative complication rate in RAMIE (59%) than open esophagectomy (80%) from a randomized controlled trial consisting of 112 patients.^23^ Yun et al also reported the significantly lower incidence of pneumonia in RAMIE as compared to open esophagectomy by a propensity score‐weighted analysis using 371 cases.^24^

In comparison between RAMIE and MIE, most of those papers showed no significant differences in the incidences of postoperative complications,^25^, ^26^, ^27^, ^28^, ^29^, ^30^ although all of them except one^30^ were retrospective studies. Harbison et al compared 100 RAMIEs and 625 MIEs from NSQIP database analysis and concluded that RAMIE might be a feasible but non‐superior option.^25^. Four propensity score‐matched studies^26^, ^27^, ^28^, ^29^ and one prospective study from a single center^30^ also reported similar results. As for lymphadenectomies, two papers reported no significant difference of the number of dissected lymph nodes,^26^, ^27^ though many papers showed the advantage of RAMIE on lymphadenectomy as compared to MIE.^28^, ^29^, ^30^, ^31^, ^32^ Especially, usefulness of the dissection along recurrent laryngeal nerve was reported to be yielded by RAMIE.^29^, ^30^, ^31^, ^32^ Yang et al noted a higher incidence of nerve injury as well as more harvested lymph nodes.^31^ As for the operation time of RAMIE, various results, i.e., longer,^26^, ^30^ similar,^27^ and shorter^31^ were observed as compared to MIE. Van Hillegersberg et al discussed, in a review article, that differences between RAMIE and MIE with respect to postoperative complications and oncological outcomes might be marginal.^33^ Ongoing randomized controlled trials comparing RAMIE to MIE^34^, ^35^ should be anticipated and examined to confirm RAMIE's benefit, including oncological outcomes. During the initial stage of induction of robot‐assisted esophagectomy, the learning curve of 26‐80 cases 36, 37, 38 should be fully recognized and modular step‐up training was proposed.^39^ Considering those factors is quite important to perform the procedures safely.

In a short summary, the superiority of RAMIE to MIE still remains controversial, though the advantage of lymphadenectomy, especially along recurrent laryngeal nerve, has been suggested. Learning curve is important at the introduction of RAMIE. Currently, robot‐assisted transmediastinal radical esophagectomy was reported to show better QOL as compared to open esophagectomy in both retrospective and prospective studies.^40^, ^41^

## 4 CENTRALIZATION TO HIGH‐VOLUME CENTER

Since hospital volume is the greatest effect to reduce operative mortality in esophagectomy among various procedures was observed by Birkmeyer et al,^42^ centralization of esophageal cancer surgery has been advancing.^43^, ^44^ Many papers published to date have also supported that effect worldwide.^8^, ^43^, ^44^, ^45^, ^46^, ^47^ Furthermore, postoperative complication rate^44^, ^45^ and operation time^44^ were reported to be reduced by centralization. However, what a high‐volume center is, remains unsolved. The number of esophagectomies per year is likely to define the high‐volume, though it was reported to vary from five to 20.^8^, ^43^, ^44^, ^46^ A population‐based, nationwide Swedish cohort study showed the superiority of university hospital to non‐university hospital status.^48^ Toh et al reported the significance of the medical institutional structure of board‐certified training sites and the participation of board‐certified surgeons based on the National Clinical Database in Japan.^49^ Another question, whether hospital or surgeon volume is more important,^50^ is also difficult to answer. Kaupplia et al reported that individual surgeon volume had a tendency to reduce mortality, but this was not showed as statistically significance.^48^

The aim of centralization is undoubtedly to offer its benefit to patients.

Riele et al pointed out that institutional characteristics had a stronger influence on mortality than volume.^51^ Cooke, also, presented important consideration. The quote is as follows; to improve the outcomes, we either must develop methods to facilitate access to centralized, high‐volume centers, or we translate the institutional knowledge, best practice and recovery and rescue pathways from our centralized programs to the communities.^52^

In a short summary, centralization to high‐volume centers evidently benefits esophageal cancer patients by improving short‐term outcomes. However, the definition of a high‐volume center has not yet been established. Regardless, the system's clinical resources and support, including manpower, are essential to aid patients, irrespectively of hospital volume, when critically adverse events occurs. Considering patient access to high‐volume centers, and sharing the knowledge and practice between high‐ and not‐high‐volume centers, is also imperative.

## DISCLOSURE

Conflict of interest: The author declares no conflict of interest for this article. 
